# Increased ACL direct insertion coverage provided more positive biomechanical effects on graft and bone tunnel during knee flexion: a simulation study

**DOI:** 10.1186/s40634-023-00677-x

**Published:** 2023-10-28

**Authors:** Yang Xiao, Zhaoxin Liang, Shiwen Shen, Fei Liu, Hai Hu, Bin Chen

**Affiliations:** 1 Division of Orthopaedics and Traumatology, Department of Orthopaedics, Nanfang Hospital, Southern Medical University, Guangzhou, China; 2 The First Clinical College of Southern Medical University, Guangzhou, China; 3 Department of Orthopedic Surgery and Orthopedic Biomechanical Laboratory, Shanghai Jiao Tong University Affiliated Sixth People’s Hospital, Shanghai, China

**Keywords:** Anterior cruciate ligament reconstruction, Direct insertion, Femoral tunnel, Finite element analysis, Biomechanics

## Abstract

**Purpose:**

Flattened femoral tunnels were recently applied in anatomical single-bundle anterior cruciate ligament (ACL) reconstruction. Little is known about the biomechanical effect of such changes during knee flexion. The aim of the present simulation study was to assess the effect of altered ACL direct insertion coverage on the biomechanics of the graft and bone tunnel.

**Methods:**

Five finite element (FE) models, including a round femoral tunnel and four progressively flattened rounded rectangular femoral tunnels, were established to represent the ACL reconstructions. In vivo knee kinematics data obtained from the validated dual fluoroscopic imaging techniques controlled the FE models to simulate lunge motions. The maximal principal stress of the graft and the volume of equivalent strain within 1000–3000 microstrain (V_1000-3000_) of the cancellous bone were subsequently calculated at 0°, 30°, 60° and 90° of knee flexion.

**Results:**

A lower stress state on the graft and a more beneficial strain state on the cancellous bone were observed when the femoral tunnel better covered the ACL direct insertion. The average maximal principal stress of each model were 3.93 ± 0.60 MPa, 3.82 ± 0.54 MPa, 3.43 ± 0.44 MPa, 3.45 ± 0.44 MPa and 3.05 ± 0.43 MPa, respectively. The average V_1000-3000_ of the cancellous bone of each model were 179.06 ± 89.62 mm^3^, 221.40 ± 129.83 mm^3^, 247.57 ± 157.78 mm^3^, 282.74 ± 178.51 mm^3^ and 295.71 ± 162.59 mm^3^, respectively. Both the stress and strain values exhibited two peaks during the flexion simulation. The highest value occurred at 30° of flexion, and the second highest value occurred at 90° of flexion.

**Conclusions:**

Increased ACL direct insertion coverage provided more positive biomechanical effects after anatomical single-bundle ACL reconstruction during knee flexion.

## Introduction

In recent years, anatomical studies have shown that the native ACL femoral footprint includes direct and indirect insertion [14, 19]. Direct insertion is located in the anterior region of the footprint, consisting of dense collagen fibres directly attached to the bone. Indirect insertion is located in the posterior area of the footprint near the femoral articular cartilage, consisting of membrane-like tissue [8, 22]. The direct fibres from the ribbon-like direct insertion bore more than 80% of the total anterior ACL load during stability testing [18]. The single-bundle ACL reconstruction using a round femoral tunnel seems to not restore the insertion sites and the fibre arrangement of the native ACL entirely [13].

Since the anatomy of the native ACL is the basis for anatomical ACL reconstruction, investigators tried to change the shape of the femoral tunnel, such as rounded rectangular and rectangular [5, 16, 25, 27]. These tailoring tunnels were flatter than the traditional round tunnel, which were expected to better restore the insertion site and morphology of the native ACL [13]. To date, two potential benefits of using a flatter femoral tunnel in anatomical single-bundle ACL reconstruction exist. Firstly, cadaver experiments showed that a flattened femoral tunnel improves the stability of the knee joint [7, 24]. Secondly, an animal experiment showed that a flattened femoral tunnel accelerates tendon-bone healing in the early period after ACL reconstruction [32].

Based on the evidence above, changing the morphology of the ACL graft will affect its function biomechanically and biologically. Theoretically, different widths of ACL direct insertion result in different graft morphology and biomechanical behaviour. There is no doubt that knee joint flexion–extension activity is closely related to complications after ACL reconstruction, such as graft injury and bone tunnel enlargement [15, 30]. Understanding the biomechanical differences caused by surgical variation is imperative for postoperative rehabilitation. The strategy of joint motion should follow anatomical and biomechanical principles. However, previous biomechanical research on changing the tunnel diameter was mainly performed under in vitro static conditions. In multiple studies, graft volume, tunnel orientation and tibial tunnel shape were inconsistent and challenging to compare.

Finite element (FE) analysis is a powerful tool for assessing the biomechanics of the knee joint [1, 28, 29]. The advantage of such analysis is that it can combine in vivo kinematic data for joint motion simulation. Moreover, it can iteratively change the geometries while keeping the other parameters constant. This is impossible in cadaver experiments. This study aimed to evaluate the biomechanical effect of changing the coverage of ACL direct insertion via FE simulations. The hypothesis of this study was that increased ACL direct insertion coverage could improve the biomechanics of graft and bone tunnel during knee flexion.

## Materials and methods

### Surgical modelling

**Fig. 1:**
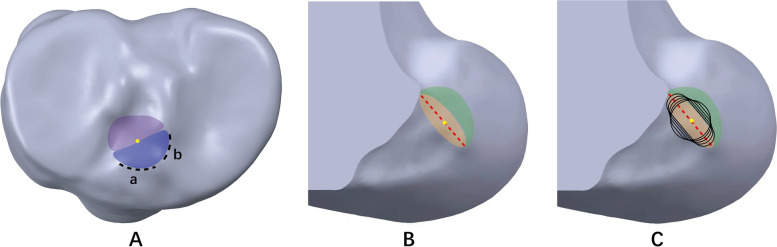
**A** Identification of the tibial footprint of the ACL (*a* anterior ridge; *b* medial intercondylar ridge; *purple region* posterolateral bundle of the ACL; *blue region* anteromedial bundle of the ACL; *yellow point* tibial attachment point). **B** Identification of the femoral footprint of the ACL (*orange region* direct ACL insertion; *green region* indirect ACL insertion; *red line* lateral intercondylar ridge; *yellow point* femoral attachment point). **C** Positional relationship among the tunnels and the landmarks on the ACL femoral footprint

The right knee of a healthy volunteer (male; 26 years; body height 175 cm; body weight 65 kg; no history of knee injuries and systemic diseases) was imaged using computed tomography (CT) (SOMATOM Definition AS + ; Siemens) with a thickness of 0.6 mm. Three-dimensional models of the femur and tibia were calculated from the CT images in Mimics (v19.0, Materialize NV, Leuven, BE). The anterior ridge and medial intercondylar ridge were identified and used to determine the ACL tibial footprint on the tibial model. The tibial attachment point was identified as the midpoint of the double bundle of the ACL on the tibial footprint (Fig. 1A) [1]. The lateral intercondylar ridge was identified and used to determine the ACL direct insertion on the femoral model [31]. The femoral attachment point was the midpoint of the lateral intercondylar ridge, near the centre of the ACL direct insertion (Fig. 1B).

**Fig. 2:**
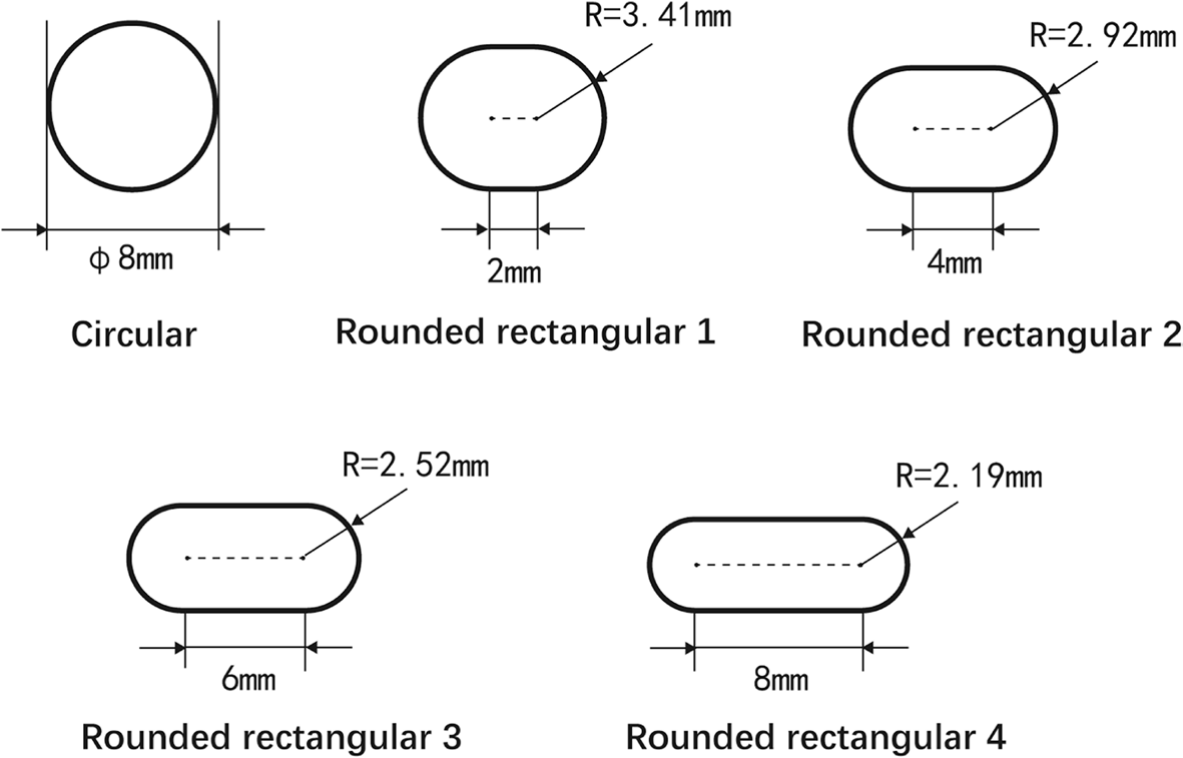
Sizes of the various tunnel apertures created in the femur

The tibial tunnel orientation was made at 15° in the front view and 55° in the sagittal view. The tibial tunnel was a traditional round tunnel with a diameter of 8 mm. The femoral tunnel orientation was made through the anteromedial portal technique, perpendicular to the medial surface of the lateral condyle. The femoral tunnel was first created as a traditional round tunnel with a diameter of 8 mm. Then, the tunnel's diameter was gradually adjusted with the central point unchanged to create four rounded rectangular femoral tunnels parallel to the lateral intercondylar ridge. The long-axis diameter of the femoral tunnel was the only variable. The tunnel with the largest long-axis diameter completely covered the direct insertion of the ACL (Fig. 1C). The sizes of the various femoral tunnel apertures are shown in Fig. 2.

**Fig. 3:**
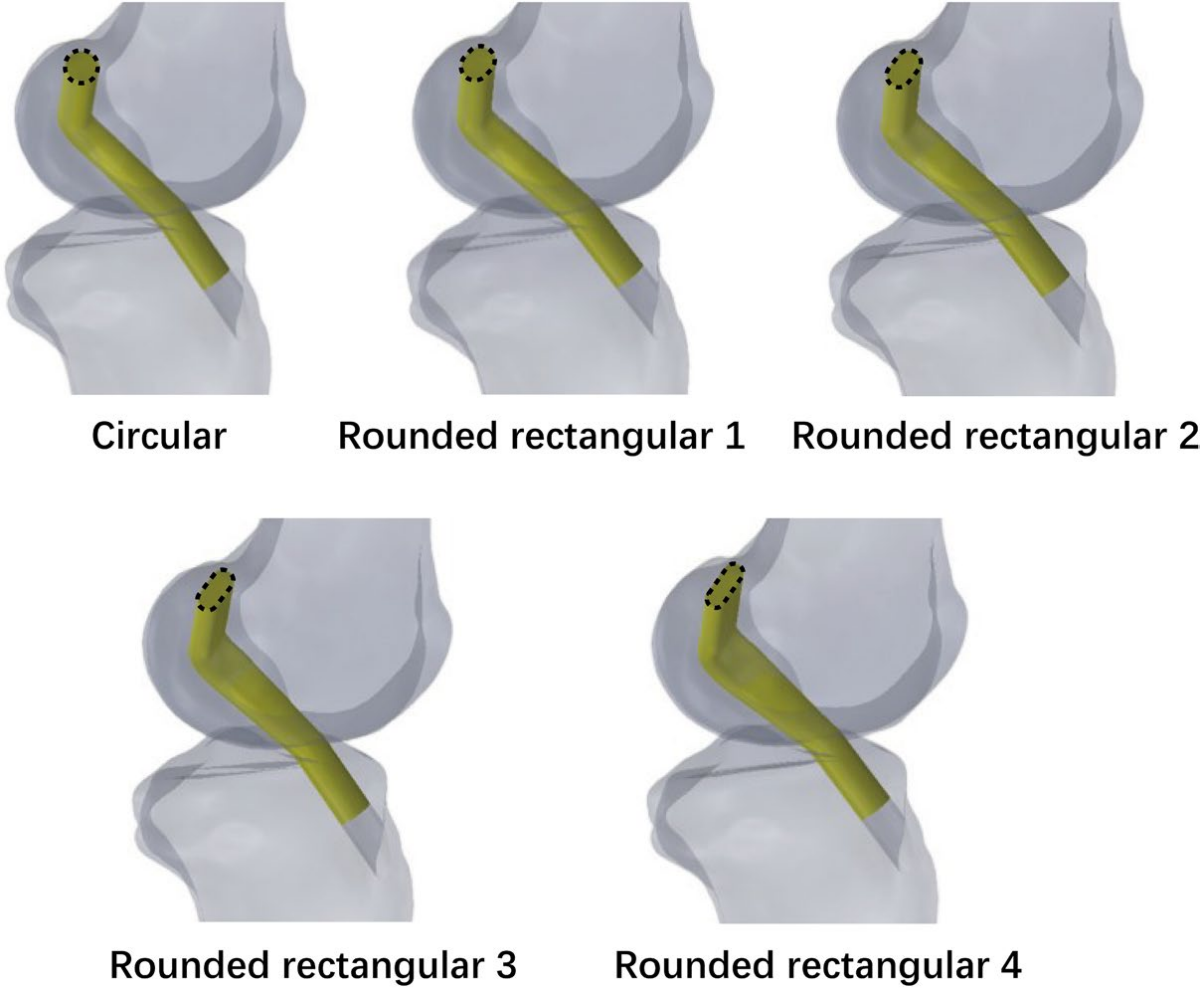
Morphology of different grafts after ACL reconstruction with different femoral tunnels

The length of the rectangular region was set as 2 mm, 4 mm, 6 mm and 8 mm. The radius of the rounded rectangle was calculated using the following equation: $S=L\times 2R+\pi \times {R}^{2}$, where the constant cross sectional area $S=\pi \times ({\frac{8}{2})}^{2}$, $L$ is the length of the rectangular region and $R$ is the radius of the rounded rectangle. The grafts measured 20 cm in depth into the femoral and tibial tunnels and were constructed as single, soft cylindrical solids (Fig. 3). All modelling procedures were performed using SolidWorks (v2018, Dassault Systemes, Massachusetts, USA).

### In vivo kinematic data

Methods of kinematic data acquisition and coordinate system (CS) establishment were the same as the previous study [29]. The experimental processes are shown as follows. First, the knee of the subject was simultaneously imaged by two fluoroscopes (BV Pulsera; Philips) when a lunge motion was performed. Then, the fluoroscopic images were positioned in the imaging planes based on the projection geometry of the fluoroscopes in MATLAB (R2013a; MathWorks). Next, local femoral CS (floating CS) and tibial CS (fixed CS) were established on the CT-based models of the femur and tibia. The models were independently manipulated in six degrees of freedom (6DOF) to match the outlines of the fluoroscopic images (30°, 60° and 90° knee flexion). Finally, the 6DOF changes in knee flexion were calculated according to the rotation matrix (floating CS relative to fixed CS) using MATLAB after the matching procedure.

### FE models of the knee joint

Simulations were performed in Abaqus (v2018; Dassault Systèmes SE, Vélizy-Villacoublay, FR). To accurately simulate the material properties of bone tunnels, the femur consisted of cortical (2 mm thick from the outer bone surface) and cancellous bones [28]. The tibia and cortical bone of the femur were considered shells and defined as rigid bodies. The cancellous bone of the femur was defined as isotropic linear elastic with Young’s modulus E = 389 MPa and Poisson’s ratio v = 0.3 [4]. The grafts were defined as nearly incompressible, transversely isotropic hyperelastic neo-Hookean materials based on the following strain-energy function: $\Psi$=$\frac{1}{2D}$ ln(*J*)^2^ + *C*_1_(${\overline{{I }_{1}}}^{2}-3$) + *F*_2_($\lambda$), where* J* is the determinant of the deformation gradient tensor, $\overline{{I }_{1}}$ is the first invariant of the modified Cauchy-Green tensor, *F*_2_ is the isochoric part of the strain-energy function depending on the collagen fibres, $\lambda$ is the stretch of the fibre, *C*_1_ is the neo-Hookean constant, and *D* is the inverse of the bulk modulus k = 1/*D* (*C*_1_ = 1.95, *D* = 0.00683) [20].

A free meshing technique was used for the cortical bone and the tibia (four-node 3D bilinear rigid quadrilateral element; element type: R3D4), the cancellous bone (four-node linear tetrahedron element; element type: C3D4) and the graft (eight-node linear hexahedral elements with hourglass control; element type: C3D8RH). A mesh sensitivity analysis was performed by stepwise upsizing the mesh size [21]. The convergence tolerance was set as a stress variation within 5% from the previous model with higher mesh density. The mesh size of grafts was between 1.0 and 1.2 mm. The mesh size of cancellous bone was 1.4 mm, and mesh refinement was applied to 1.0 mm around the tunnel entrances. The graft and cancellous bone models included an average of 4000-plus nodes with 3000-plus elements and 57,000-plus nodes with 306,000-plus elements, respectively.

### Boundary and loading conditions & simulation outputs

**Fig. 4:**
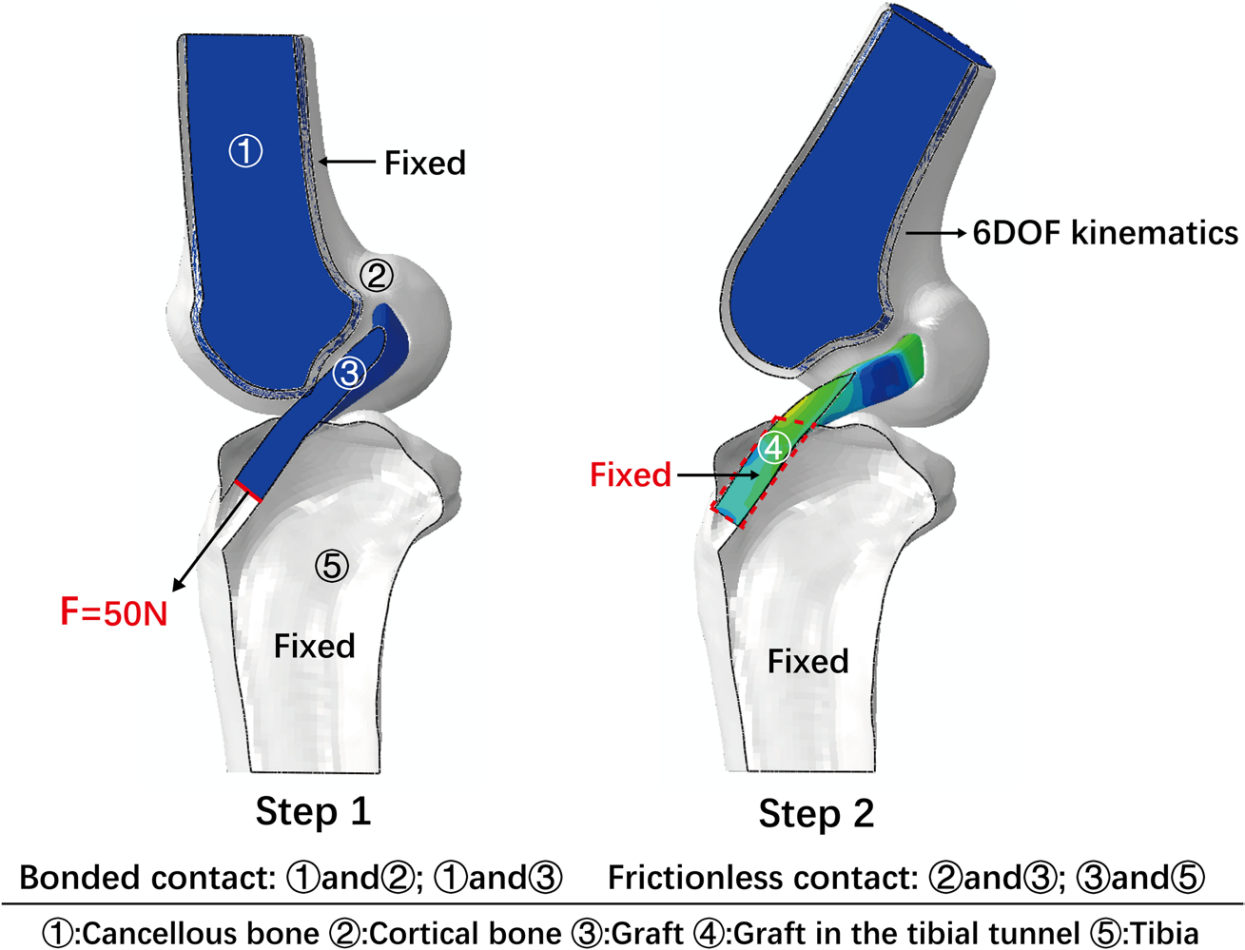
The constraint and load conditions applied in the simulation

Bonded contact between the cortical bone and the cancellous bone was defined. Bonded contact between the graft and the internal surface of the femoral cancellous bone tunnel was defined. Frictionless contact between the graft and tibial tunnel and between the graft and femoral cortical bone tunnel was defined as suggested by a previous study [28]. Two steps of loading conditions were implemented. First, the femur and tibia were fixed, and an initial graft tension of 50 N was applied on the end surface of the graft along the tibial tunnel direction. Second, the tibia and the portion of the graft in the tibial tunnel were fixed, and the femur was translated and rotated according to the 6DOF calculation relative to the femoral CS described above (Fig. 4).

**Fig. 5:**
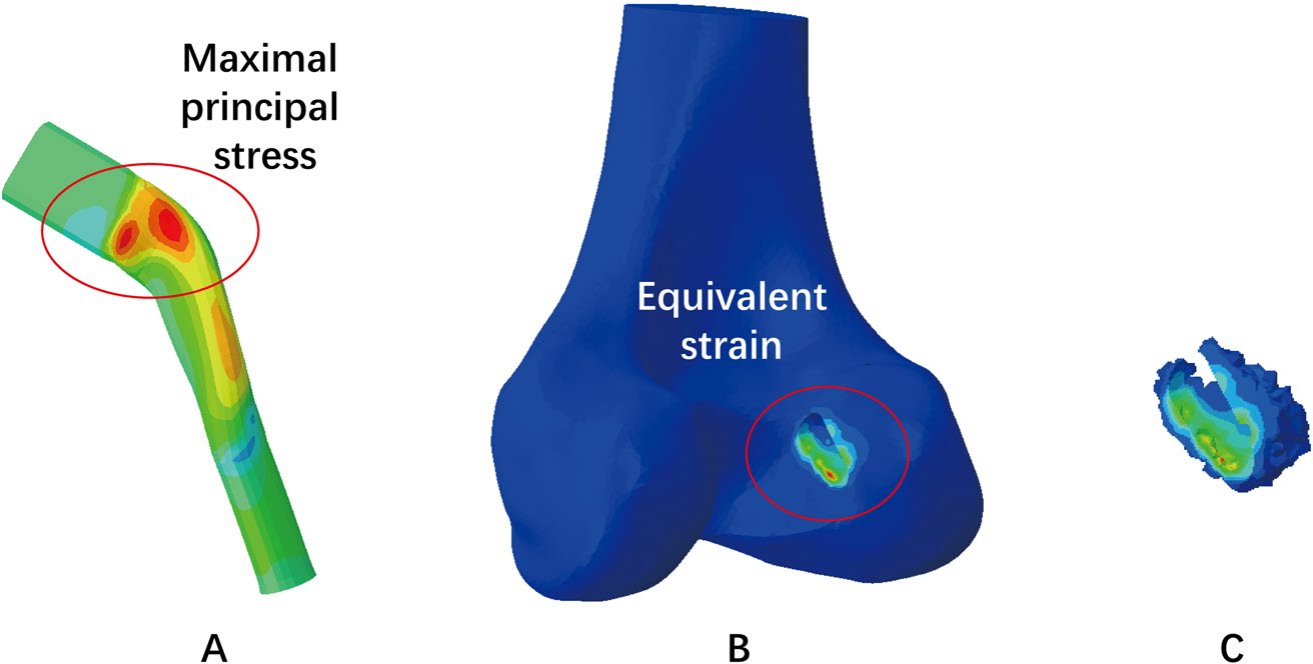
**A** The maximal principal stress on the graft was calculated for each model during flexion simulation; **B** The equivalent strain on the cancellous bone around the tunnel entrances; **C** The volume of equivalent strain within 1000–3000 microstrain on the cancellous bone was recorded

The maximum principal stress on the graft and the equivalent strain of the cancellous bone around the tunnel entrances were selected as the biomechanical indicators and subsequently calculated. The strain range in the 1000–3000 microstrain region was considered beneficial for bone modelling [6]. The equivalent strain was defined as $\varepsilon =\sqrt{\frac{1}{2}[{\left({\varepsilon }_{1}-{\varepsilon }_{2}\right)}^{2}+{\left({\varepsilon }_{1}-{\varepsilon }_{3}\right)}^{2}+{\left({\varepsilon }_{2}-{\varepsilon }_{3}\right)}^{2}]}$, where ${\varepsilon }_{1}$, ${\varepsilon }_{2}$ and ${\varepsilon }_{3}$ are three principal strains [12]. The volume of equivalent strain within 1000–3000 microstrain (V_1000-3000_) on the cancellous bone was recorded (Fig. 5).

## Results

### Maximal principal stress

**Fig. 6:**
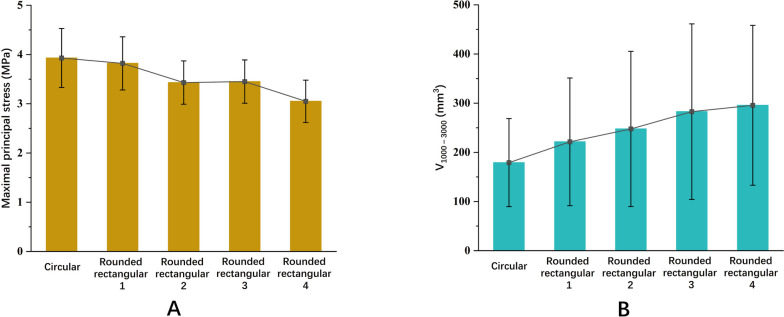
**A** Comparison of the average maximal principal stress on the graft among the different tunnel sizes during knee flexion. **B** Comparison of the average V_1000-3000_ on cancellous bone among the different tunnel sizes during knee flexion

The maximal principal stress of the graft appeared near the entrance to the femoral tunnel at all flexion angles. Two peaks of stresses were found during the flexion simulation. The highest value occurred at 30° of flexion, and the second highest value occurred at 90° of flexion. Compared to the round tunnel, decreased maximal principal stresses were found at all angles when the tunnel size became flattened. Through the whole range of motion, the average maximal principal stress of each model was 3.93 ± 0.60 MPa, 3.82 ± 0.54 MPa, 3.43 ± 0.44 MPa, 3.45 ± 0.44 MPa and 3.05 ± 0.43 MPa, respectively (Fig. 6A; Table 1).

**Table 1:** The maximal principal stress (MPa) of the graft with different femoral tunnel sizes at various flexion angles

Flexion angle	Circular	Rounded rectangular 1	Rounded rectangular 2	Rounded rectangular 3	Rounded rectangular 4
0°	3.37	3.36	3.01	2.97	2.89
30°	4.45	4.44	4.02	4.00	3.69
60°	3.45	3.37	3.20	3.28	2.74
90°	4.45	4.10	3.50	3.55	2.87

### Equivalent strain

**Fig. 7:**
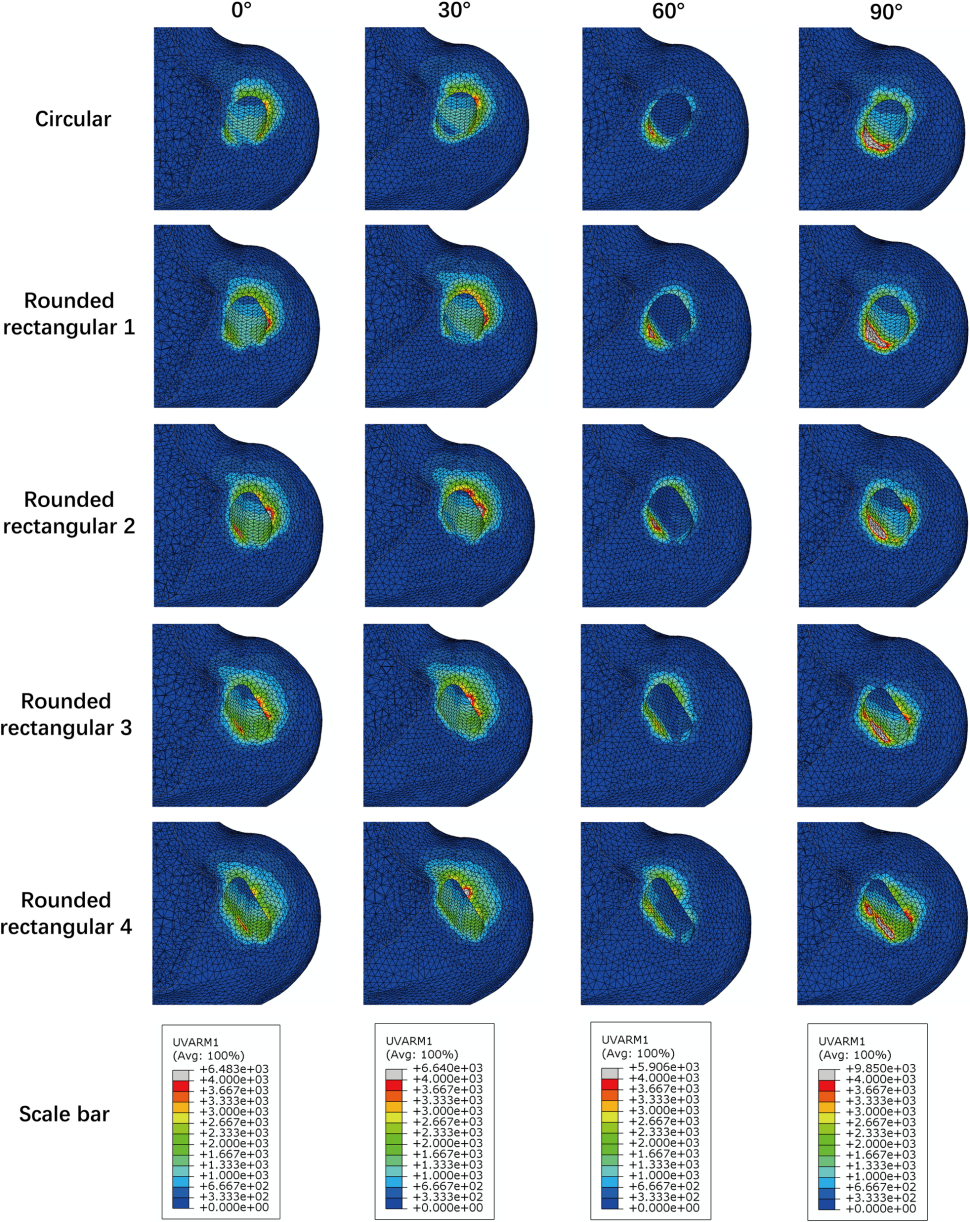
Distribution of equivalent strain on the cancellous bone around the tunnel entrances

The distribution of equivalent strain on the cancellous bone for each model is displayed in Fig. 7.

The V_1000-3000_ on the cancellous bone has two peaks during the flexion simulation. The largest V_1000-3000_ occurred at 30° of flexion, and the second largest V_1000-3000_ occurred at 90° of flexion. Increased V_1000-3000_ values were found when the tunnel size generally became flattened. Through the whole range of motion, the average V_1000-3000_ of the cancellous bone of each model was 179.06 ± 89.62 mm^3^, 221.40 ± 129.83 mm^3^, 247.57 ± 157.78 mm^3^, 282.74 ± 178.51 mm^3^ and 295.71 ± 162.59 mm^3^, respectively (Fig. 6B; Table 2).

**Table 2:** The volume of equivalent strain within 1000–3000 microstrain (mm^3^) on the cancellous bone with different femoral tunnel sizes at various flexion angles

Flexion angle	Circular	Rounded rectangular 1	Rounded rectangular 2	Rounded rectangular 3	Rounded rectangular 4
0°	187.47	288.66	348.24	365.92	340.63
30°	261.42	350.11	399.38	479.27	478.24
60°	52.68	51.73	51.95	68.94	86.84
90°	214.67	195.09	190.71	216.84	277.14

## Discussion

The most important finding of the present study is that increasing the ACL direct insertion coverage positively affects the biomechanics of the graft and bone tunnel after anatomical single-bundle ACL reconstruction during knee flexion. Graft stresses and strains around the bone tunnels can explain these findings. From the perspective of the flexion angle, the maximal principal stress of the graft and the V_1000-3000_ of cancellous bone around the tunnel increased first. Then it decreased from 0° to 60° of flexion when the maximum force was reached at 30° of flexion. Both values increased when the knee joint was at 90° of flexion. From the perspective of tunnel diameter, changing the femoral tunnel size had obvious biomechanical differences. Increased ACL direct insertion coverage results in larger V_1000-3000_ of cancellous bone on the femoral tunnel entrances and a lower maximal principal stress on the ACL graft.

Previous biomechanical studies have clarified that direct fibres lead to higher loads than indirect fibres when restraining anterior tibial translation and rotation [10, 18]. Considering that traditional ACL reconstruction using a round femoral tunnel breaks the native ACL attachment and histologic structures, it may be necessary to use reasonable tunnel-shaping methods to overcome this limitation of the surgery from the anatomical and biomechanical point of view. Recently, a biomechanical study of cadaveric specimens demonstrated that both single bundle ribbon-like and round grafts used in ACL reconstruction restored the kinematics of the intact knee at time zero [3]. Since the recovery of the knee joint after ACL reconstruction takes time, the differences in graft stress and bone tunnel strain caused by tunnel sizes may be potential factors affecting long-term clinical outcomes.

Orthopaedic surgeons have used the rounded rectangular femoral tunnel in primary or revision ACL reconstruction, yielding good clinical outcomes [7, 17, 23]. The long-axis diameters of the rounded rectangle dilator varied from 9 to 12 mm in surgery. Different femoral tunnels determine the different morphologies and volumes of grafts, which may affect the results of surgical reconstruction. A rounded rectangular femoral tunnel led to an anatomical femoral insertion with a flat tendon graft, providing better fibre arrangement and resembling the original ACL. Moreover, stress concentration on the graft was thought to be closely related to graft injuries after surgery [9]. This study indicated that the maximal principal stress on the graft is near the entrance of the femoral tunnel, which is the most frequent location of graft failure in autograft [11]. The results also showed that the trends of change in maximal principal stress of the graft decreased when the tunnel became flatter. Thus, increasing the long-axis diameter of the femoral tunnel when the graft volume is constant may represent an effective strategy in anatomical single-bundle ACL reconstruction.

Bone tunnel enlargement is a common phenomenon after ACL reconstruction caused by mechanical and biological factors [30]. The bone tunnel and reconstructed ligament undergo repetitive mechanical changes with knee joint extension and flexion. A suitable mechanical environment is beneficial to bone modelling and tendon-bone healing. A previous study demonstrated that the strain value inside the bones was an essential factor determining bone formation or resorption in response to mechanical loading [6]. The bone modelling process is switched when bone strains exceed 1000 microstrain. Repeated bone strains cause microscopic fatigue damage in bone with an operational threshold strain range greater than 3000 microstrain [6]. Researchers also used the strain around the bone tunnel to account for bone tunnel enlargement after ACL reconstruction in FE simulation [2]. The current results showed that the volume of cancellous bone strain within 1000–3000 microstrain increased as the tunnel flattened. A previous study showed that the CT value of the femoral bone tunnel was significantly higher for the rounded rectangular tunnel than the round tunnel [26]. This observation was consistent with the current results, as increasing the long-axis diameter of the femoral tunnel in ACL reconstruction might be beneficial to bone modelling as it results in a lower tunnel enlargement ratio.

This study has several limitations. Only a few flexion angles were selected, and the entire range of motion was not analysed. Because the knee joint is activity-dependent, other functional activities, such as walking and ascending stairs, may cause different biomechanical environments at the bone tunnel and the ACL graft. In addition, rigid contact between the graft and bone tunnel was applied in the simulation with no consideration of relative motion occurring between the graft and the tunnel wall, which was not entirely consistent with clinical practice. Furthermore, the present study was conducted with a subject-specific model. The tunnel sizes in this study included the maximum width of clinical application but not the maximum width of other individuals. Further study should consist of other subjects and tunnel sizes to determine whether this is a typical result.

## Conclusions

The results of this study confirm that increased ACL direct insertion coverage provided a more positive effect during knee flexion after anatomical single-bundle ACL reconstruction, resulting in a lower stress state on the ACL graft and a more beneficial strain state of cancellous bone on femoral tunnel entrances. The present study is meaningful as it provides biomechanical evidence for clinical practice.

## Data Availability

The data that support the findings of this study are available from the corresponding author upon reasonable request.

## Abbreviations

ACL: Anterior cruciate ligament
FE: Finite element
CT: Computed tomography
CS: Coordinate system
6DOF: Six degrees of freedom
V_1000-3000_: Volume of equivalent strain within 1000–3000 microstrain
